# HLA-B^*^58:01 for Allopurinol-Induced Cutaneous Adverse Drug Reactions: Implication for Clinical Interpretation in Thailand

**DOI:** 10.3389/fphar.2016.00186

**Published:** 2016-07-18

**Authors:** Chonlaphat Sukasem, Thawinee Jantararoungtong, Parnrat Kuntawong, Apichaya Puangpetch, Napatrupron Koomdee, Patompong Satapornpong, Patcharin Supapsophon, Jettanong Klaewsongkram, Ticha Rerkpattanapipat

**Affiliations:** ^1^Division of Pharmacogenomics and Personalized Medicine, Department of Pathology, Faculty of Medicine Ramathibodi Hospital, Mahidol UniversityBangkok, Thailand; ^2^Laboratory for Pharmacogenomics, Somdech Phra Debaratana Medical Center, Ramathibodi HospitalBangkok, Thailand; ^3^The Thai Severe Cutaneous Adverse Drug Reaction Research GroupBangkok, Thailand; ^4^Department of Pharmacy, Somdech Phra Debaratana Medical Center, Ramathibodi HospitalBangkok, Thailand; ^5^Division of Allergy and Clinical Immunology, Department of Medicine, Faculty of Medicine, Allergy and Clinical Immunology Research Group, Chulalongkorn UniversityBangkok, Thailand; ^6^Division of Allergy Immunology and Rheumatology, Department of Medicine, Faculty of Medicine Ramathibodi Hospital, Mahidol UniversityBangkok, Thailand

**Keywords:** HLA-B^*^58:01, allopurinol, Thai, SJS, TEN, DRESS, MPE, drug hypersensitivity

## Abstract

**Background:** The aim of this study was to investigate the predisposition to different types of allopurinol-induced cutaneous adverse drug reactions (CADR), including Stevens-Johnson syndrome (SJS), toxic epidermal necrolysis (TEN; SJS-TEN, *n* = 13), drug reaction with eosinophilia and systemic symptoms (DRESS, *n* = 10) and Maculopapular eruption (MPE; *n* = 7), conferred by HLA-B^*^58:01 in a Thai population.

**Methods:** This case-control association study compares 30 patients with allopurinol-induced CADR, allopurinol-tolerant control patients (*n* = 100), and a Thai general population (*n* = 1095). Patients' *human leukocyte antigen type B (HLA-B)* alleles were genotyped by using a two-stage sequence-specific oligonucleotide probe system.

**Results:** Of a total 30 patients with CADR due to allopurinol, 29 (96.7%) patients were found to be at least heterozygous for HLA-B^*^58:01, compared to only 4.0% in allopurinol-tolerant patients (*p* < 0.001). Odds ratio (OR) for the association of HLA-B^*^58:01 with allopurinol-induced CADR in this population was 696.0 (95% CI: 74.8–6475.0). The HLA-B^*^58:01 allele was present in all patients with allopurinol-induced SJS-TEN (OR = 579.0, 95%CI: 29.5–11362.7, *p* < 0.001) and DRESS (OR 430.3, 95%CI: 22.6–8958.9, *p* < 0.001). Additionally, OR of HLA-B^*^58:01 was highly significant in the allopurinol-induced MPE patients (OR 144.0, 95%CI: 13.9–1497.0, *p* < 0.001).

**Conclusion:** In this study we confirmed the association between HLAB^*^58:01 and allopurinol-induced SJS-TEN in a Thai population. In addition, we identified an association between HLA-B^*^58:01 and allopurinol-induced DRESS and MPE in this population. Therefore, HLA-B^*^58:01 can be used as a pharmacogenetic marker for allopurinol-induced CADR including SJS-TEN, DRESS and MPE. These results suggest that screening for HLA-B^*^58:01 alleles in patients who will be treated with allopurinol would be clinically helpful in preventing the risk of developing CARD in a Thai patients.

**Summary**

- Regardless of phenotype, this is the first pharmacogenetic study of allopurinol-induced CADR in patients of Thai ancestry.
- In this study we confirmed the association between HLA-B^*^58:01 and allopurinol-induced SJS-TEN, DRESS, and MPE in Thai population.
- Regarding to our findings, the pharmacogenetic interpretation could be generalized to drug hypersensitivity including DRESS, SJS-TEN, and MPE.

## Introduction

In this last decade, pharmacogenetic studies have shown a strong association between *human leukocyte antigen (HLA)* alleles and susceptibility to drug hypersensitivity reactions (Sukasem et al., 2014). *HLA* genes are a major contributor to drug hypersensitivity involving direct stimulation of immune effector cells and imitating an allergic reaction (Adam et al., 2011; Cheng and Su, 2014). Currently, *HLA-B* alleles have been used as pharmacogenetic markers to predict drug-induced cutaneous adverse drug reactions (CADR; Daly, 2014; Sukasem et al., 2014). The CADR such as Stevens-Johnson syndrome (SJS), toxic epidermal necrolysis (TEN), drug reaction with eosinophilia and systemic symptoms (DRESS) or drug hypersensitivity syndrome (DHS), and acute generalized exanthematous pustulosis (AGEP) are often life-threatening. Maculopapular eruption (MPE) is recognized as a mild form of hypersensitivity reaction (Lonjou et al., 2008; Karlin and Phillips, 2014; Sasidharanpillai et al., 2015).

Clinical presentation of SJS and TEN is characterized by rapid progression of mucosal detachment and systemic symptoms which may present as fever, mild elevation of hepatic enzymes, and intestinal and pulmonary manifestation (Barvaliya et al., 2011; Harr and French, 2012). SJS and TEN are differentiated by the seriousness of skin detachment, which is limited in SJS (< 10% of body surface area; BSA) and more widespread in TEN (>30% of BSA), with the intermediate stage (10–30% of BSA) of skin detachment referred to as SJS/TEN overlapping (Aihara, 2011; Harr and French, 2012). DRESS syndrome is an extremely serious adverse effect referred to sometimes as DHS. It is characterized by a skin rash, lymphadenopathy, fever, and can involve single or multiple organs (Aihara, 2011; Fleming and Marik, 2011).

Allopurinol is a commonly prescribed medication that has been used to inhibit xanthine oxidase in patients with gouty arthritis, hyperuricemia, and in cancer patients undergoing chemotherapy (Lam et al., 2013; Min et al., 2015). In Thailand, allopurinol is a major cause of CADR and has been reported as the second most frequent cause of CADR, including SJS-TEN (SJS, TEN, SJS/TEN) and DRESS (Tassaneeyakul et al., 2009; Saokaew et al., 2014). Several studies have reported that severe reactions to allopurinol are strongly associated with HLA-B^*^58:01, which is carried by 8–15% of Han Chinese and Thais (Hung et al., 2005; Tassaneeyakul et al., 2009; Puangpetch et al., 2015), but occurs relatively infrequently in Japanese (0.6%) and European (0.8%) populations (Kaniwa et al., 2008; Lonjou et al., 2008; Goncalo et al., 2013). According to the data from the spontaneous reports by the Health Product Vigilance Center of Thailand, allopurinol is the second ranked of common culprit drugs, with at least 1488 patients suffering from SJS-TEN and at least 75 patients suffering from DRESS during the last 20 years (http://thaihpvc.fda.moph.go.th/thaihvc/Public/News/uploads/hpvc_5_13_0_100526.pdf).

A previous publication from Thailand showed that the HLA-B^*^58:01 allele is a strong marker for allopurinol-induced CADR in the Thai population (Tassaneeyakul et al., 2009). However, that study reported only an association between allopurinol-induced SJS-TEN and HLA-B^*^58:01. More recently, a high frequency of HLA-B^*^58:01 was reported in Portuguese patients with allopurinol-induced DRESS also (Goncalo et al., 2013). Therefore, this study aims to determine the association of allopurinol-induced CADR, which includes DRESS and SJS-TEN, and also MPE, with the HLA-B^*^58:01 allele in Thai patients.

## Materials and methods

### Subjects and characteristics

In this study, we carried out research as a retrospective and prospective case-control study. From 2011 to 2015, patients with allopurinol-induced CADR admitted to the allergy clinic of Faculty of Medicine Ramathibodi Hospital, Mahidol University were enrolled. Thirty patients with allopurinol-induced CADR were categorized into DRESS (10 cases), SJS-TEN (13 cases) and MPE (7 cases). Patients who had been taking allopurinol for more than 6 months without evidence of cutaneous adverse effects were recruited as allopurinol-tolerant controls (*n* = 100). In addition, general population who had not taken allopurinol and had no history of drug induced cutaneous adverse reactions were included in this study. Both case and control subjects were independently recruited with no family relationship. Data for this healthy control group was obtained from 1095 subjects undergoing *HLA-B* genotyping through the Laboratory for Pharmacogenomics, Somdech Phra Debaratana Medical Center (SDMC), Ramathibodi Hospital, Thailand.

The study was performed and approved by the Ramathibodi Hospital ethical review board, and informed consent was obtained from all of the participants.

### Diagnosis of cutaneous adverse drug reactions (CADR)

All CADR patients were assessed by a dermatologist and allergist who reviewed photographs, pathological slides, clinical morphology, and medical records. The diagnosis of drug-induced DRESS, SJS-TEN was made according to the RegiSCAR criteria (Choudhary et al., 2013). In brief, DRESS was diagnosed in patients presenting with fever, maculopapular rash with internal organ involvement, and hematologic abnormalities. SJS was diagnosed in patients with skin rash and mucosal erosion covering up to 10% of BSA whereas SJS-TEN overlap was diagnosed in patients with epidermal necrosis whose blistering skin lesions affected between 10 and 30% of BSA. MPE was diagnosed in patients presenting with danger signs in drug-induced exanthema or covering 30% BSA with or without associated systemic symptoms, but not fulfilling criteria for DRESS (Pichler et al., 2002).

### Genomic DNA extraction

Blood samples were collected into EDTA tubes. DNA was isolated using the MagNA Pure automated extraction system (Roche Diagnostics, USA) based on magnetic-bead technology. The quality of genomic DNA was assessed using a Nano Drop ND-1000 to measure quantity and purity of genomic DNA. All DNA was aliquotted and stored at −20°C before analysis.

### *HLA-B* typing

The *HLA-B* alleles were genotyped by the Polymerase Chain Reaction-sequence specific oligonucleotide probe (PCR-SSOP) principles with the commercial kit (LABType SSO HLA Typing Kit; One Lambda Inc., CA, USA). Then, the *HLA-B* alleles were carried out using Luminex™ Multiplex Technology (Luminex® IS 100, USA). Briefly, PCR products were hybridized against a panel of oligonucleotide probes coated on polystyrene microspheres that have sequences complementary to stretches of polymorphic sequence within the target *HLA-B* alleles. The amplicon-probe complex was visualized using a colorimetric reaction and fluorescence detection technology. Data analysis for the *HLA-B* assays were performed with HLA fusion^TM^ 2.0 software.

### Statistical analysis

The association between HLA-B^*^58:01 and allopurinol-induced CADR was evaluated by comparing the group of individuals with CADR with the allopurinol-tolerant groups and the general population. Data were counted by presence or absence of HLA-B^*^58:01 allele. Chi-square test and Fisher's exact test were used to analyze the association between allopurinol-induced cutaneous adverse reactions and HLA-B^*^58:01. Statistical analysis was performed using SPSS version 16.0 (SPSS Inc., Chicago, IL, USA). The strength of association was estimated by calculating the odds ratio (OR) with a 95% confidence interval (CI). Sensitivity, specificity, positive predictive value (PPV) and negative predictive value (NPV) were calculated. *P* ≤ 0.05 (two-sided) were considered to indicate statistical significance.

## Results

### Subjects clinical characteristics

Of the 30 patients with CADR, 23 had underlying gout and seven had hyperuricemia. Seventeen (56.7%) patients were male and 13 (43.3%) were female, with an average age of 73.3 years (range 30–88 years). The mean duration of allopurinol use was 16.4 ± 14.3 days with a mean dosage of 239.29 ± 87.0 mg/day (range, 100–600 mg/day).The mean interval from allopurinol initiation to symptom onset was 22.2 ± 12.9 days (range, 7–42 days). The onset of symptoms for all patients was within the first 2 months of allopurinol exposure. The most common underlying were Hypertension consisting of 20 patients (66.7%) followed by Chronic Kidney Disease (CKD) with 13 patients (43.3%) and Diabetes with 4 patients (13.3%). The most common of co-medication was colchicine (19/30; 63.3%). Patient's characteristics, duration of allopurinol exposure to symptom onset and the results of *HLA-B* genotyping are summarized in Tables 1, 2.

**Table 1:** **Association of demographic data and Allopurinol-induced cutaneous adverse drug reactions (CADR)**.

**Demographic data**	**Case (*n* = 30)**
**SEX (n/%)**	
Male	17/56.7
Female	13/43.3
Age (median/range)	73/30–88
**UNDERLYING DISEASE (n/%)**	
Hypertension	20/66.7
Chronic kidney disease	13/43.3
Diabetes	4/13.3
**CO-MEDICATION**	
Colchicine	19/63.3
Simvastatin	3/10.0
Prednisolone	2/6.7
**DOSAGE OF ALLOPURINOL; mg/day**	**239.29 ± 87.0**
**(AVERAGE ± SD)**	
**DURATION OF ALLOPURINOL EXPOSURE; DAYS**	**16.4 ± 14.3**
**(AVERAGE ± SD)**	
**LABORATORY RESULT (AVERAGE ± SD)**	
Uric acid; mg/dL	6.58 ± 2.537
Blood Urea Nitrogen; mg/dL	43.64 ± 32.341
Creatinine; mg/dL	2.45 ± 5.246
Aspartate aminotransferase; mg/dL	63.19 ± 88.120
Alanine transaminase; mg/dL	71.26 ± 96.188
**PHENOTYPE (n/%)**	
SJS-TEN	13/43.4
DRESS	10/33.3
MPE	7/23.3

**Table 2:** **Summary of characteristic and genotyping data of allopurinol-induced cutaneous adverse drug reactions (CADR) in individuals**.

**No**.	**Age/Sex**	**Phenotype**	HLA-B^*^58:01	***HLA-B* genotyping**
1	56/M	DRESS	Positive	*4402:5801*
2	72/F	SJS	Positive	*5701:5801*
3	78/F	SJS	Positive	*3501:5801*
4	68/F	SJS	Positive	*4601:5801*
5	85/F	MPE	Positive	*4001:5801*
6	48/F	SJS	Positive	*1301:5801*
7	68/M	SJS	Positive	*1301:5801*
8	74/M	DRESS	Positive	*5201:5801*
9	54/M	SJS-TEN	Positive	*4001:5801*
10	78/M	SJS-TEN	Positive	*4403:5801*
11	74/F	SJS-TEN	Positive	*1502:5801*
12	28/M	DRESS	Positive	*3915:5801*
13	37/F	DRESS	Positive	*0801:5801*
14	67/M	MPE	Negative	*1301:5401*
15	81/F	DRESS	Positive	*1301:5801*
16	76/F	SJS	Positive	*5801:5801*
17	76/M	SJS	Positive	*4001:5801*
18	73/M	DRESS	Positive	*5201:5801*
19	76/F	SJS-TEN	Positive	*1513:5801*
20	73/F	SJS-TEN	Positive	*4601:5801*
21	55/M	SJS-TEN	Positive	*1502:5801*
22	55/F	DRESS	Positive	*5801:5801*
23	78/M	DRESS	Positive	*1502:5801*
24	81/M	DRESS	Positive	*5801:5801*
25	61/M	DRESS	Positive	*5101:5801*
26	88/F	MPE	Positive	*4001:5801*
27	79/M	MPE	Positive	*4001:5801*
28	73/M	MPE	Positive	*1301:5801*
29	72/M	MPE	Positive	*1802:5801*
30	84/M	MPE	Positive	*3901:5801*

### HLA-B^*^58:01 in cutaneous adverse drug reactions (CADR) case-control study

To identify genetic markers for allopurinol–induced CADR including DRESS, SJS-TEN, and MPE, we carried out a case-control association study. Frequencies of HLA-B^*^58:01 genotype in the three groups are shown in Table 2. Of the 30 patients with allopurinol-induced CADR, 29 patients (96.70%) carried HLA-B^*^58:01, while 4 of 100 (4.0%) allopurinol-tolerant controls and 111 of 1095 (10.1%) untreated controls carried this allele. The frequency of HLA-B^*^58:01 in subjects with allopurinol-induced CADR was notably higher than in the allopurinol-tolerant group (OR 696.00; 95% CI: 74.81–6475.01, *p* < 0.001) and general population group (OR 257.08; 95% CI: 34.68–1905.57). In our *HLA-B* genotyping studies, no other alleles showed significant association with allopurinol-induced CADR (Table 3).

**Table 3:** **The association of individual *HLA-B* allele with allopurinol-induced cutaneous adverse drug reactions (CADR)**.

***HLA-B* allele**	**Allopurinol-induced CADR (*n* = 30) (%)**	**Allopurinol tolerant control (*n* = 100) (%)**	**General population (*n* = 1095) (%)**	**CADR case vs. allopurinol tolerant control**		**CADR case vs. general population**	
				**OR (95% CI)**	***p*-value**	**OR (95% CI)**	***p*-value**
***58:01***	**29 (96.7)**	**4 (4.0)**	**111 (10.1)**	**696.00 (74.81–6475.01)**	**<0.001**	**257.08 (34.68–1905.57)**	**<0.001**
*13:01*	5 (16.7)	9 (9.0)	137 (12.5)	2.02 (0.62–6.58)	0.242	1.40 (0.53–3.71)	0.501
*15:02*	3 (10.0)	20 (20.0)	161 (14.7)	0.44 (0.12–1.6)	0.218	0.65 (0.19–2.15)	0.475
*40:01*	5 (16.7)	29 (29.0)	162 (14.8)	0.49 (0.17–1.40)	0.184	1.15 (0.43–3.05)	0.776
*46:01*	2 (6.7)	25 (25.0)	227 (20.7)	0.21 (0.05–0.96)	0.051	0.27 (0.06–1.16)	0.077
*51:01*	1 (3.3)	12 (12.0)	65 (5.9)	0.25 (0.03–2.03)	0.196	0.55 (0.07–4.08)	0.556

By extending our investigation for other HLA-B alleles, we found that HLA-B^*^40:01 (29.0%, OR = 0.45; 95% CI, 0.09–2.13), HLA-B^*^46:01 (25.0%, OR = 0.21; 95% CI, 0.05–0.96), and HLA-B^*^51:01 (12.0%, OR = 0.25; 95%CI, 0.03–2.03) was detected more frequently in control group than in allopurinol-induced CADR groups (16.7, 6.7, and 3.3%, respectively). However, there were no statistically significant differences (*p* > 0.05) between case and control groups (Table 3).

### HLA-B^*^58:01 in SJS-TEN and DRESS cases-controls study

The relationship between HLA-B^*^58:01 and allopurinol-induced SJS-TEN and DRESS was subsequently studied in this study. All 23 (100%) patients with allopurinol-induced SJS-TEN (*n* = 13) and DRESS (*n* = 10) cases had HLA-B^*^58:01 (three patient was homozygous for HLA-B^*^58:01). As shown in Table 4, the *HLA-B 58:01* allele occurred at significantly increased frequencies among the allopurinol-induced SJS-TEN patients compared to the two control groups (OR = 579.00, 95%CI: 29.50–11362.67 and OR = 238.40, 95%: 14.08–4037.80). Sensitivity and specificity of HLA-B^*^58:01 for prediction of allopurinol-induced SJS-TEN were 100.00 and 96.0%. In addition, the PPV and NPV of the HLA-B^*^58:01 allele was also 76.47 and 100.0%, respectively (Table 7).

**Table 4:** **The association of individual *HLA-B* allele with allopurinol-induced SJS-TEN**.

***HLA-B* allele**	**Allopurinol-induced SJS-TEN (*n* = 13) (%)**	**Allopurinol tolerant control (*n* = 100) (%)**	**General population (*n* = 1095) (%)**	**SJS-TEN cases vs. Allopurinol tolerant control**		**SJS-TEN cases vs. general population**	
				**OR (95% CI)**	***p*-value**	**OR (95% CI)**	***p*-value**
***58:01***	**13 (100.0)**	**4 (4.0)**	**111 (10.1)**	**579.00 (29.50–11362.67)**	**<0.001**	**238.40 (14.08–4037.80)**	**<0.001**
*13:01*	2 (15.4)	9 (9.0)	137 (12.5)	1.84 (0.35–9.62)	0.471	1.27 (0.28–5.80)	0.756
*15:02*	2 (15.4)	20 (20.0)	161 (14.7)	0.73 (0.15–3.55)	0.694	1.05 (0.23–4.80)	0.945
*40:01*	2 (15.4)	29 (29.0)	162 (14.8)	0.45 (0.09–2.13)	0.312	1.05 (0.23–4.77)	0.953
*46:01*	2 (15.4)	25 (25.0)	227 (20.7)	0.55 (0.11–2.63)	0.450	0.70 (0.15–3.16)	0.638
*51:01*	0 (0.0)	12 (12.0)	65 (5.9)	0.26 (0.01–4.69)	0.363	0.58 (0.03–9.91)	0.709

In addition, the HLA-B^*^58:01 allele was associated with a higher risk of DRESS (OR 430.33, 95%CI: 22.64–8958.88, *p* < 0.001 and OR 185.42, 95%CI: 10.79–3185.84, *p* < 0.001) when compared with allopurinol tolerant patients and the general population, respectively (Table 5). Sensitivity and specificity of HLA-B^*^58:01 for prediction of allopurinol-induced DRESS were 100.00 and 96.0%. In addition, the PPV and NPV of the HLA-B^*^58:01 allele was also 76.43 and 100.0%, respectively (Table 7).

**Table 5:** **The association of individual *HLA-B* allele with allopurinol-induced DRESS**.

***HLA-B* allele**	**Allopurinol-induced DRESS (*n* = 10) (%)**	**Allopurinol tolerant control (*n* = 100) (%)**	**General population (*n* = 1095) (%)**	**DRESS cases vs. allopurinol tolerant control**		**DRESS cases vs. general population**	
				**OR (95% CI)**	***p*-value**	**OR (95% CI)**	***p*-value**
***58:01***	**10 (100.0)**	**4 (4.0)**	**111 (10.1)**	**430.33 (22.64–8958.88)**	**<0.001**	**185.42 (10.79–3185.84)**	**<0.001**
*13:01*	1 (10.0)	9 (9.0)	137 (12.5)	1.12 (0.13–9.90)	0.917	0.78 (0.10–6.18)	0.812
*15:02*	1 (10.0)	20 (20.0)	161 (14.7)	0.44 (0.05–3.72)	0.454	0.64 (0.08–5.12)	0.678
*40:01*	1 (10.0)	29 (29.0)	162 (14.8)	0.27 (0.03–2.25)	0.225	0.64 (0.08–5.08)	0.673
*46:01*	0 (0.00)	25 (25.0)	227 (20.7)	0.14 (0.01–2.49)	0.181	0.18 (0.01–3.11)	0.240
*51:01*	1 (10.0)	12 (12.0)	65 (5.9)	0.82 (0.09–7.01)	0.852	1.76 (0.22–14.11)	0.594

### HLA-B^*^58:01 in MPE cases-controls study

Of the seven patients with allopurinol-induced MPE, 6 of 7 (85.7%) patients had HLA-B^*^58:01 whereas 4 (4.0%) of allopurinol-tolerant patients had HLA-B^*^58:01. The one patient with MPE who did not have the HLA-B^*^58:01 allele carried HLA-B^*^13:01/54:01. In this study, the OR of HLA-B^*^58:01 was highly significant in the allopurinol-induced MPE patients (OR 144.00, 95%CI: 13.85–1497.03, *p* < 0.001), as shown in Table 6. Sensitivity and specificity of HLA-B^*^58:01 for prediction of allopurinol-induced MPE were 85.71 and 96.0%, respectively. In addition, the PPV and NPV of the HLA-B^*^58:01 allele was also 60.0 and 98.97%, respectively (Table 7).

**Table 6:** **The association of individual *HLA-B* allele with allopurinol-induced MPE**.

***HLA-B* allele**	**Allopurinol-induced MPE (*n* = 7) (%)**	**Allopurinol tolerant control (*n* = 100) (%)**	**General population (*n* = 1095) (%)**	**MPE case vs. Allopurinol tolerant control**		**MPE case vs. general population**	
				**OR (95% CI)**	***p*-value**	**OR (95% CI)**	***p*-value**
***58:01***	**6 (85.7)**	**4 (4.0)**	**111 (10.1)**	**144.00 (13.85–1497.03)**	**<0.001**	**53.19 (6.35–445.85)**	**<0.001**
*13:01*	2 (28.6)	9 (9.0)	137 (12.5)	4.04 (0.68–23.91)	0.123	2.80 (0.54–14.56)	0.222
*15:02*	0 (0.0)	20 (20.0)	161 (14.7)	0.26 (0.01–4.77)	0.366	0.39 (0.02–6.79)	0.515
*40:01*	3 (42.9)	29 (29.0)	162 (14.8)	1.84 (0.39–8.72)	0.445	4.32 (0.96–19.48)	0.057
*46:01*	0 (0.0)	25 (25.0)	227 (20.7)	0.20 (0.01–3.58)	0.272	0.25 (0.01–4.47)	0.349
*51:01*	0 (0.0)	12 (12.0)	65 (5.9)	0.47 (0.03–8.78)	0.615	1.05 (0.06–18.57)	0.974

**Table 7:** **Sensitivity/Specificity/Positive predictive value (PPV)/Negative predictive value (NPV)**.

HLA-B^*^58:01	**vs. allopurinol tolerant control (%)**				**vs. general population (%)**			
	**Sensitivity**	**Specificity**	**PPV**	**NPV**	**Sensitivity**	**Specificity**	**PPV**	**NPV**
CADR	96.67	96.00	87.88	98.97	96.67	89.86	20.71	99.90
SJS-TEN	100.00	96.00	76.47	100.00	100.00	89.86	10.48	100.00
DRESS	100.00	96.00	76.43	100.00	100.00	89.86	8.26	100.00
MPE	85.71	96.00	60.00	98.97	85.71	89.86	5.13	99.90

## Discussion

In the present study, the case–control analysis included 30 cases of allopurinol-induced CADR, which included DRESS (10 cases), SJS-TEN (13 cases), and MPE (7 cases). The association study in Thai patients examined only a limited phenotype of allopurinol-induced SJS-TEN (Tassaneeyakul et al., 2009). In this study we confirmed the association between HLA-B^*^58:01 and allopurinol-induced SJS-TEN (OR = 579.0) in a Thai population. In addition, we identified an association between HLA-B^*^58:01 and allopurinol-induced DRESS and MPE with OR 430.3 and 144.0, respectively. Thus, the HLA-B^*^58:01 is associated with allopurinol-induced CADR including SJS-TEN, DRESS and MPE in a Thai population.

HLA-B^*^58:01 was the most predominant allele associated with allopurinol-induced CADR and was not found in only one patient who had allopurinol-induced MPE (HLA-B^*^13:01/54:01). Moreover, there was no significantly associated CADR with any other *HLA-B* alleles such as HLA-B^*^13:01, indicating that HLA-B^*^58:01 has an important role in the progression of allopurinol-related CADR in the Thai population. The odds ratio (OR) for the association of HLA-B^*^58:01 with combined CADR phenotypes in this population was 696.00 (*p* < 0.01). Using the allopurinol tolerant group as the control, the HLA-B^*^58:01 allele had 95.20% sensitivity and 100% specificity for diagnosing CADR. This strong association also has been observed in other Asian countries (Hung et al., 2005; Kaniwa et al., 2008; Jung et al., 2011; Kang et al., 2011).

Previous study has proposed association of immune mechanisms in the development of several forms of allopurinol-induced CADR. Hung et al. has shown that the HLA-B^*^58:01 allele is a strong genetic factor in the incidence of CADR (SJS-TEN, and DRESS) for Han Chinese taking allopurinol (Hung et al., 2005). Although, Tassaneeyakul et al. was the first to identify an association between HLA-B^*^58:01 and allopurinol-induced SJS-TEN in Thai (Tassaneeyakul et al., 2009), no published data have yet confirmed such a strong correlation of HLA-B^*^58:01 and allopurinol-induced DRESS and MPE in Thai patients. Recently, we identified an association between HLA-B^*^58:01 and allopurinol-induced DRESS and MPE in Thai population.

This finding reveals that the risk of developing DRESS among those allopurinol users with HLA-B^*^58:01 is significantly increased by 430.3 times compared to allopurinol-tolerant controls. The association is 100% in that the HLA-B^*^58:01 was present in all 10 patients with allopurinol-induced DRESS, similar to the study in Han Chinese and Japanese populations (Hung et al., 2005; Kaniwa et al., 2008). In this study, we also confirmed a strong association between the allele HLA-B^*^58:01 and susceptibility to allopurinol-induced SJS-TEN in Thai patients. Based on the strong association of the presence of HLA-B^*^58:01 and DRESS and SJS-TEN, it is presumed that the attributable risk of CADR due to the existence of this allele is larger—as high as 8% in Thailand (Puangpetch et al., 2015)—indicating that HLA-B^*^58:01 is associated with the pathogenesis of allopurinol-induced CADR regardless of the phenotype or severity. This is in contrast to carbamazepine-induced SJS-TEN, with which HLA-B^*^15:02 only shows association with only SJS-TEN (Suresh Kumar et al., 2005; Phillips et al., 2011; Lee et al., 2014).

Moreover, MPE has been considered to be distinct from SJS-TEN, characterized as macule and papule rash, with symmetry on both left and right of body and especially on the face, palms and feet, and with no detachment on the body surface area and no systemic involvement (Pichler et al., 2002). The association of HLA-B^*^58:01 with MPE is less well studied (Profaizer and Eckels, 2012). Recently, Cao et al. found that all 22 Han Chinese with MPE in that study were HLA-B^*^58:01 positive (Cao et al., 2012). Among 12 Australian patients, none was HLA-B^*^58:01 positive, and three of four had MPE, which was statistically significantly different from the allopurinol-tolerant group (Lee et al., 2012). In recent times, we investigated an association between HLA-B^*^58:01 and allopurinol-induced MPE. By comparison, the OR of HLA-B^*^58:01 was 144.0 and 53.2 between the MPE patients with the allopurinol-tolerant and untreated Thai population groups. However, a major limitation of this study was the sample size of the MPE patients available for the analysis. Confirmation of this result in an independent cohort of larger sample size would allow us to determine whether the HLA-B^*^58:01 identified in this study is definitely associated with the development of allopurinol-induced MPE and provide more accurate estimates of their impact in the clinical practice.

Commonly, allopurinol is associated with CADR ranging from mild skin rash, such as MPE to life-threatening severe cutaneous adverse reactions including DRESS and SJS-TEN (Ng et al., 2016). Interestingly, HLA-B^*^40:01 was found much more frequently in the allopurinol-induced MPE (*n* = 3/7; 42.9%) than others allopurinol-induced SJS-TEN (*n* = 2/13; 15.4%) and no HLA-B^*^40:01 was observed in allopurinol-induced DRESS cases. Furthermore, homozygous HLA-B^*^58:01 was found only in severe cutaneous adverse drug reaction, that included SJS-TEN and DRESS. Hence, the complementary alleles with HLA-B^*^58:01 carriers might be identified as a marker influencing susceptibility to different types of allopurinol-induced CADR in Thai population. Moreover, Grover et al. found that HLA-B^*^40:01 could be a protective marker for carbamazepine-induced CADR (OR = 0.32; 95% CI = 0.19–0.53; *P* = 1.08 × 10^−5^; Grover and Kukreti, 2013). In this study, HLA-B^*^40:01 (29.0%), HLA-B^*^46:01 (25.0%), and HLA-B^*^51:01 (12.0%) was higher in allopurinol-tolerant than in allopurinol-induced CADR groups (16.7, 6.7, and 3.3%, respectively, *p* > 0.05). This possibly suggests that the both alleles might be protective markers for allopurinol-induced CADR. However, the number of patients may not be enough to reveal all the assumptions, further investigation using a large number of samples and well-designed study is required to better understand.

Practically, colchicine is an immune-modulating agent which is normally prescribed with allopurinol for acute gout prophylaxis. Ryu et al. found that the use of colchicine was the clinical risk factor for adverse events when using allopurinol (Ryu et al., 2013). In this study, 63.3% (*n* = 19/30) of patients with allopurinol-induced CADR were treated with colchicine as a co-medication. Both allopurinol and colchicine are potential offending drugs in the present case of CADR. Commonly, allopurinol, one of the most frequent causes of SJS and TEN. Colchicine is also associated, but to a lesser degree (Ryu et al., 2013). However, the risk of allopurinol-induced CADR in Concurrent administration with colchicine in patients carried HLA-B^*^58:01 allele has not yet been evaluated.

**Figure 1:**
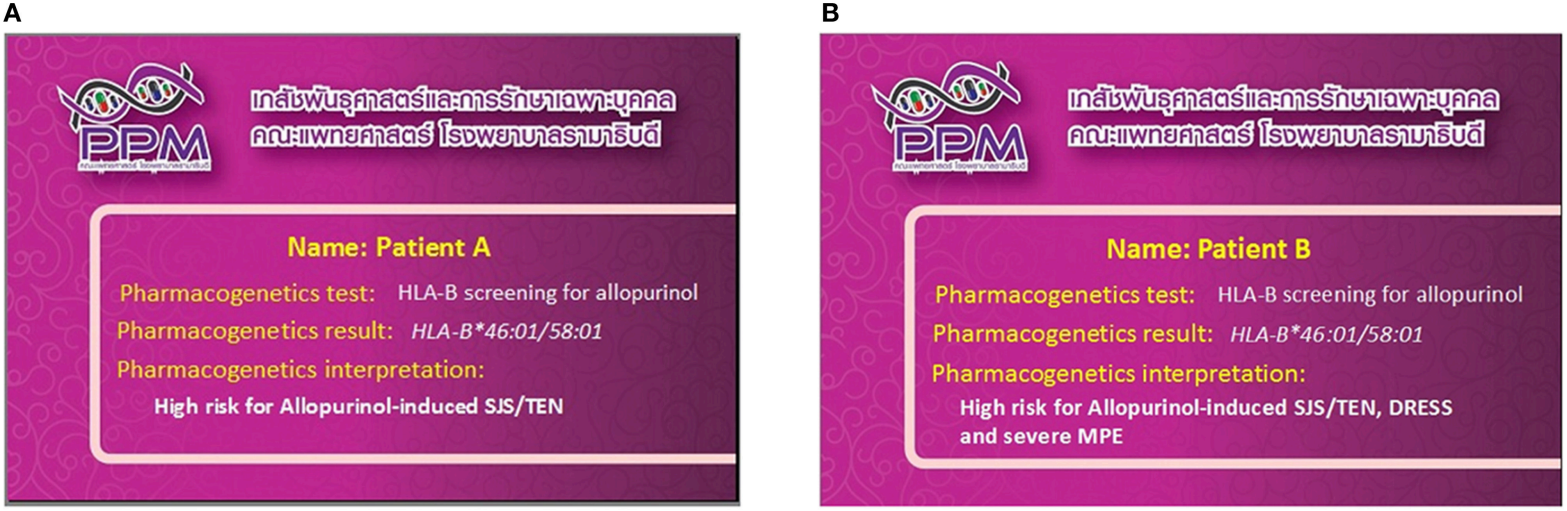
**Pharmacogenomics card and clinical interpretation; (A) clinical interpretation before this study, (B) revised clinical interpretation after this study**.

With evidence support the association of the HLA-B^*^58:01 allele with allopurinol-induced CADR, CPIC guidelines recommend the use of pharmacogenomics tests for presence of the HLA-B^*^58:01 allele before initiating allopurinol therapy in patients (Hershfield et al., 2013). After patients have taken an HLA test, their results are entered into a plastic “pharmacogenomic wallet card,” which basically contains the genomic results of those related to the risk of CADR. This card can be carried around and shown to different doctors in the future (Figures 1A,B). Currently, there were over 1400 patients which were genotyped and delivered the pharmacogenomic cards for screening HLA-B^*^58:01 before allopurinol prescription from our setting. The pharmacogenetics interpretation has been changed from “High risk for allopurinol-induced SJS-TEN” (Figure 1A) to be “High risk for allopurinol-induced SJS-TEN, DRESS and MPE” (Figure 1B) from our finding in Thai population. Physicians and national policy makers should consider genetic screening for the HLA-B^*^58:01 alleles prior to initiation of allopurinol therapy in Thai patients.

In summary, a strong association between allopurinol-induced CADR and the HLA-B^*^58:01 allele was confirmed. Incidence of the HLA-B^*^58:01 allele is strongly associated with individuals who are at risk for allopurinol-induced DRESS, SJS-TEN, and MPE in the Thai population. Our results suggest that the screening tests for the HLA-B^*^58:01 allele in patients who will be treated with allopurinol would be clinically helpful in reducing the risk of developing CADR.

## Author contributions

CS designed and operated project, set goals and controlled project, analyzed results, and supervised the pharmacogenetic section. TJ assisted to coordinate project between pharmacogenetic part and clinical part. PK collected samples and extracted genomic DNA. AP analyzed statistical data. NK performed HLA typing. PS collected samples and performed HLA typing. PS managed clinical part and counseled all patients. JK controlled project, evaluated the effectiveness of treatment and advised the methodology. TR co-designed and co-operated project, controlled the operations to meet the goal, managed clinical part, and counseled all patients.

### Conflict of interest statement

The authors declare that the research was conducted in the absence of any commercial or financial relationships that could be construed as a potential conflict of interest.

## Abbreviations

AGEP: Acute generalized exanthematous pustulosis
BSA: Body surface area
CADR: Cutaneous adverse drug reactions
DRESS: Drug reaction with eosinophilia and systemic symptoms
DHS: Drug hypersensitivity syndrome
HLA: Human leukocyte antigen
MPE: Maculopapular eruption
SJS: Stevens-Johnson syndrome
TEN: Toxic epidermal necrolysis
PPV: Positive predictive value
NPV: Negative predictive value.
